# The effects of online pilates on the mood and fear of birth of pregnant women: a randomized controlled study

**DOI:** 10.1038/s41598-024-67290-5

**Published:** 2024-07-12

**Authors:** Merve Bulguroglu, Halil I. Bulguroglu

**Affiliations:** Faculty of Health Sciences, Department of Physiotherapy and Rehabilitation, Ankara Medipol University, Ankara, Turkey

**Keywords:** Online exercise, Pilates, Depression, Fear of birth, Health care, Medical research

## Abstract

The Pilates exercise method is applied online to various population groups. This study aimed to determine the effect of online Pilates exercises on depression, anxiety, and fear of childbirth in pregnant women. Our randomized controlled study divided participants into the online pilates group (OPG) and the control group (CG). Pilates exercises were applied to the OPG according to the American College of Obstetricians and Gynecologists guidelines, while the CG was given a home program. Assessments were made before and after the training. All measurements improved after Online Pilates (*p* < 0.05), while there was no change in the control group (*p* > 0.05). While initial measurement values were similar in both groups (*p* > 0.05), a statistical difference was observed in favor of OPG with a moderate effect percentage in all results after training (*p* < 0.05). These results revealed that eight weeks of online Pilates training could effectively reduce depression, anxiety, and fear of childbirth.

**Trial registration:**Clinical trial registry: NCT05305716.

## Introduction

Pregnancy, one of the critical periods in women’s lives, is a natural physiological event, but it also may bring about physiological stress reactions in the body^1^. Thus, pregnancy is a physiological stress situation that affects all body systems and requires the woman’s physical, mental, and social harmony^2^. During pregnancy, worries about pregnancy and the following periods, such as “the thought that some risks that may arise during childbirth may harm the baby,” can cause stress, anxiety, and depression and affect the healthy progress of pregnancy^3^. Women may fear childbirth^4^. Fear of childbirth in mothers can trigger other symptoms, such as depression. Pregnant women with depression are known to have more pregnancy-related symptoms, such as pain and insomnia^5^. As the pregnancy progresses, experienced problems, emotional changes, and the consequent activity limitations may cause a decrease in the general quality of life^6^.

The World Health Organization reported that women minimize their physical activity levels during pregnancy due to concerns that exercise may harm the pregnancy and the fetus^7^. Changes during pregnancy may also lead to decreased physical activity levels^8^. Pandemic processes such as COVID-19, which has affected the entire society, have also reduced the participation of pregnant women in physical activities, primarily face-to-face physical activities^9^. During pregnancy, women may be more willing to follow health promotion recommendations and make related lifestyle changes^10^. From this point of view, this period can also be considered a period of opportunity for women to gain healthy living habits. In order to have a healthy pregnancy, the mother needs to manage her body both physically and mentally. Some practices that can be done during pregnancy can increase the mother’s adaptation to the changes in pregnancy. Exercise is the most important of these^11^.

The guide published by the American College of Obstetricians and Gynecologists states that pregnant women should do at least half an hour of moderate-intensity exercise every day of the week. This guideline also recommended Pilates, yoga, and swimming as safe activities for pregnant women^12^. Pilates has proven effects on pregnant women as well as on the entire population. Pilates does not reduce blood flow to the fetus and helps relieve the stress that pregnancy brings to the body. Most importantly, it makes women feel more comfortable in this quick process and makes women more active. Therefore, Pilates is a protective and effective exercise system for mothers throughout pregnancy^13–17^. These benefits of Pilates should be continued under all circumstances.

Telerehabilitation, which has become one of the prominent fields in recent years, reduces the obstacles to rehabilitation services, such as time, cost, and distance, by providing rehabilitation services by experts using computer-based technologies and communication tools^18^. Especially during the COVID-19 pandemic, we have experienced online exercise applications. This technology-based telerehabilitation method has helped almost everyone spend their time at home more efficiently and maintain healthy habits. One of the online exercise applications used is the Pilates exercise method. The effectiveness of online Pilates applications in different populations has been examined in various studies, and it has been stated that they have different advantages and disadvantages. In common, studies have shown that online Pilates exercise is at least as safe and effective as face-to-face practice in most cases^19–24^. Studies regarding the effectiveness of the online method during pregnancy are not considered sufficient in the literature. Two online studies on Pilates during pregnancy found that pregnant women reduced their physical activity. However, in both studies, fear of birth and anxiety experienced during pregnancy, which are perhaps the most critical problems of pregnant women, were not evaluated. Mood changes during pregnancy can also negatively affect the pregnant woman physically. In addition, it is noteworthy that the effects of exercise, especially in the prenatal period, have not been adequately examined in the studies, and the number of participants is low^25,26^. Therefore, this study aims to understand how the depression, anxiety, and fear of childbirth levels of pregnant women who do Pilates online are affected and to bring a new approach to telerehabilitation.

## Methods

### Study design and population

Before starting our study, which we conducted at Ankara Medipol University, Faculty of Health Sciences, Department of Physical Therapy and Rehabilitation, ethical approval was received from the Gazi University Ethics Committee with research code 2021–1037. Our study was conducted per CONSORT 2010 guidelines and regulations (Clinical Trials.gov Number NCT05305716, registration date 31/03/2022) and the Declaration of Helsinki.

Fifty-eight pregnant women were invited to our randomized controlled study with the guidance of obstetricians and gynecologists. Inclusion criteria: Primiparous and singleton pregnancy was defined as being between 20 and 35, being at least in the 16th week of pregnancy, and volunteering as exclusion criteria; exclusion of heart and lung problems, all situations in which exercise is contraindicated during pregnancy, and participation in different activity programs.

After five people withdrew to participate in the study, fifty-three were randomly assigned after an initial assessment to the Online Pilates group (OPG; = 27) or the control group (CG; *n* = 26) by a simple method (Microsoft Excel, 2016). The evaluator and statistical analyzer were blind to group assignment. Before the study, informed consent was obtained from the participants stating that the content of the study was explained and that the study was voluntary.

### Intervention

Pilates training was conducted online for one hour, two days a week for eight weeks, by Halil Ibrahim Bulguroglu, a certified and experienced Physiotherapist from the Australian Pilates and Physiotherapy Institute, using the Microsoft Teams program. Although there is no international guide for pilates exercises during pregnancy, there are simple recommendations. The Australian Pilates and Physiotherapy Institute has modified the exercises, especially in line with the recommendations of the American College of Obstetricians and Gynecologists^12^. We considered these suggestions in our study. All individuals received one session of training before the Pilates exercise training. In this training, the pregnancy process, what the individual expects during this process, why he/she should exercise, what Pilates training is, its goals, and why it is an appropriate exercise method for pregnant women were explained, and the key elements of Pilates are; breathing, focusing, rib cage placement, centering, shoulder placement, head and neck placement were taught. During the training, individuals were asked to focus on key elements and maintain the smoothness of these elements during movements. In addition, the Walk-Talk test was explained to pregnant women, and they were told what they should do to ensure that their workload does not exceed moderate intensity. The one-hour program was arranged as a warm-up, pilates exercises, and a cool-down program. The exercise program recommended by the Australian Pilates and Physiotherapy Institute during pregnancy was used in Pilates exercises. We applied online pilates exercises to pregnant women in the same trimester and small groups of 4–5 people. The exercises were explained to the participants using verbal and visual techniques. The pilates exercise program is given in Table 1. Exercises were always applied in the same order. Posture and stretching exercises were applied during the cooling period. Exercise intensity was increased with the resistance of the elastic bands (Theraband et al. Corporation, Akron, Ohio). Resistance was increased by starting with red elastic bands and switching to blue after two weeks. If the new resistance was challenging for the pregnant woman, the exercises were continued with the same color band for another week. During the exercises, participants were informed about side effects such as shortness of breath and dizziness that they may experience and asked to stop exercising if any side effects occurred. After the first evaluation, the pregnant women in the control group were given an approximately 50-min home program consisting of breathing and relaxation exercises. They were asked to practice for one hour twice a week for eight weeks. The first applications were made online under the instructor’s supervision, and in the fourth week, they were called by phone to check whether they had completed the program.

**Table 1:** Pilates exercises program.

Movements	0–4 weeks	4–8 weeks
1. Arm scissors		
2. Windmills		
3. Pillow squeeeze		
4. Abdominal prep		
5. Gluteal stretch		
6. Buttock squeeze		
7. Bend and stretch with theraband	2 set 6–8 repetition	2 set 8–12 repetition
8. Lift and lower with theraband		
9. Clam variations		
10. Cat stretch		
11. Thread the needle		
12. Row		
13. Triceps in standing		
14. Ball push-up at the wall		

### Outcome measurements

The participants included in the study were evaluated with data collection forms filled with a questionnaire during a face-to-face interview twice before and after the training programs. The participants’ demographic information (age, body weight, height, gestational age, and body mass index) was recorded. Depression symptoms were measured with the Edinburgh Postpartum Depression Scale (EPDS)^27^. Bunevicius et al.^28^ reported in their study in 2009 that EPDS is a reliable tool for evaluating depressive symptoms during pregnancy. Other studies also use EPDS in pregnancy^29,30^. EPDS is a self-report scale comprising ten items; the lowest score obtained from the scale is 0, and the highest score is 30. On the scale with a cut-off score of 13, a score of 13 and above indicates the risk of depression. The scale’s Cronbach alpha value, which Engindeniz^31^ adapted to Turkish and which is valid and reliable, was found to be 0.87. In our study, Cronbach’s alpha value was found to be 0.791. Anxiety and Fear of Birth symptoms were secondary outcomes assessed with the State-Trait Anxiety Inventory (STAI Form 1–2)^32^ and Wijma Birth Expectation/Experience Questionnaire Version A (W-DEQ-A)^33^. The STAI has two subscales to measure two independent concepts of anxiety: the State Anxiety Scale (S-Anxiety) and the Trait Anxiety Scale (T-Anxiety) are two subscales of the STAI to measure the independent concept of anxiety. State anxiety is defined as the temporary emotional state of an organism, while Trait anxiety is understood as a stable, anxious tendency to increase anxiety. Both subscales have ‘0 items’, and the four response options range from 0 to 3. A high score indicates a high level of anxiety. Oner and Le Compte^34^ made a Turkish adaptation of the questionnaire. Cronbach’s alpha values for the Trait Anxiety Scale are between 0.83 and 0.87; for the state anxiety scale, they are between 0.94 and 0.96. In this study, Cronbach’s alpha value for the Trait Anxiety Scale was 0.82, and for the State Anxiety Scale was 0.85. The W-DEQ was developed to measure the nature of fear of childbirth by asking women questions about prenatal (version A) and postpartum (version B). Scoring on the 33-item scale is a six-point Likert type numbered from 0 to 5. While the maximum score that can be obtained from the scale is 165, a higher score indicates that women experience more fear of childbirth. The Cronbach alpha value of the scale, adapted to Turkish by Korukcu and Kukulu^35^ and has validity and reliability, was found to be 0.89. In our study, Cronbach’s alpha value was found to be 0.91.

### Statistical analysis

The statistical analysis using SPSS version 21 (SPSS et al., USA) determined the significance level as *p* < 0.05 in all tests. Normal distribution was determined using histograms, probability plots, and the Shapiro–Wilk test. Median and interquartile range (IQR) were used because descriptive statistics showed an non-normal distribution. The Mann–Whitney U test was used for demographic characteristics, while within-group values at baseline and after eight weeks were compared with the Paired Sample t-test. An Independent sample t-test was used to compare measurement results between the study and control groups. In our study, effect sizes were also evaluated according to Cohen’s standards. Effect sizes were interpreted as small (≥ 0.2), medium (≥ 0.5), or large (≥ 0.8)^36^.

## Results

**Figure 1:**
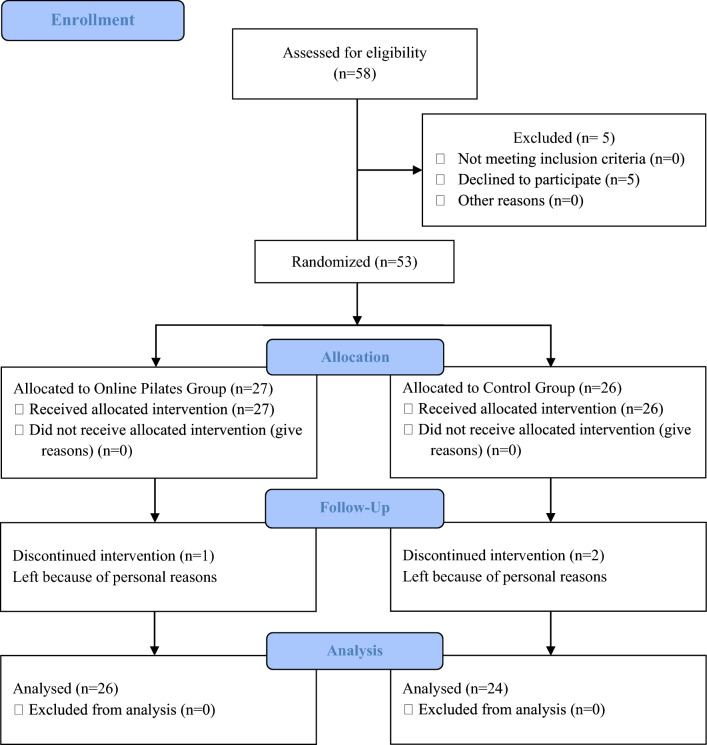
Flow diagram outlining participation involvement.

One person in the Online Pilates group and two in the control group did not participate in the final evaluation for personal reasons. The study was completed with 26 participants from OPG and 24 from CG among the 58 pregnant women invited within the scope of the study. Figure 1 shows the participants’ CONSORT flowchart. No adverse effects were observed in any of our participants during our study. When the post hoc power analysis of the study was calculated, taking depression values into account, the effect size was found to be 1.213. With 26 participants in OPG and 24 participants in CG, the power of the study was calculated as 0.86.

While the demographic characteristics of all participants are shown in Table 2, there was no difference between the groups regarding these variables (*p* > 0.05).

**Table 2:** Demographic characteristics of the groups.

	OPG Median (IQR) *n* = 26	CG Median (IQR) *n* = 24	*p*
Age (years)	27 (25–31)	28 (27–30)	0.149
Height (cm)	168 (155–179)	167 (157–176)	0.512
Weight (kg)	71 (53–95)	69 (54–96)	0.432
Gestational age at study start (weeks)	17 (14–20)	16 (13–19)	0.753
BMI-1 (kg/cm^2^)	23.34 (19.93–32.78)	23.89 (20.46–28.97)	0.741
BMI-2 (kg/cm^2^)	26.91 (19.84–32.81)	27.58 (20.34- 29.97)	0.246

*p < 0.05. *OPG* Online pilates group; *CG* Control group; *cm* centimeters, *kg* kilograms, *BMI* Body mass index.

Although the exercises were performed remotely online, there was an improvement in depression, anxiety, and fear of birth levels in OPG in terms of initial and final values (*p* < 0.05, Table 3). At the same time, there was no statistical difference in all parameters between the initial and final values in CG (*p* > 0.05, Table 3). While there was no difference between the initial depression, anxiety, and fear of birth values in both groups (*p* > 0.05, Table 4), a statistically significant difference was observed with a moderate effect percentage in favor of the Online Pilates group in all parameters in the measurement values after training (*p* < 0.05, Table 4).

**Table 3:** Comparison of depression, anxiety and fear of childbirth levels of groups before and after training.

	Group	Before training X ± SD	After training X ± SD	*p*^1^	Effect size
EPDS (0–30)	OPG	8.23 ± 1.98	5.32 ± 1.84	0.02*	1.02
	CG	7.67 ± 1.94	7.79 ± 1.32	0.36	0.12
WDEQ-A (0–165)	OPG	61.73 ± 18.96	49.87 ± 20.16	0.01*	0.97
	CG	59.89 ± 11.23	58.16 ± 15.5	0.67	0.21
State-Trait Anxiety Inventory (20–80)					
State anxiety	OPG	35.13 ± 7.66	31.18 ± 5.84	0.04*	0.65
	CG	36.62 ± 3.13	36.93 ± 5.03	0.83	0.07
Trait anxiety	OPG	36.67 ± 3.91	31.98 ± 2.21	0.01*	0.81
	CG	34.37 ± 2.20	35.15 ± 3.71	0.55	0.23

*p < 0.05; ^1^: paired samples t-test; *X* Mean; *SD* Standard Deviation; *OPG* Online Pilates group; *CG* Control group; *EPDS* Edinburgh Postnatal Depression Scale; *WDEQ-A* Wijma Delivery Expectancy/Experience Questionnaire A.

Significant values are in bold.

**Table 4:** A comparison of the previous and subsequent measurement of depression, anxiety and fear of childbirth for pilates and control groups.

		OPG (mean ± SD)	CG (mean ± SD)	*p*^2^	Effect size
EPDS (0–30)	Before Training	8.23 ± 1.98	7.67 ± 1.94	0.065	0.82
	After Training	5.32 ± 1.84	7.79 ± 1.32	0.041*	0.70
WDEQ-A (0–165)	Before Training	61.73 ± 18.96	59.89 ± 11.23	0.087	0.21
	After Training	49.87 ± 20.16	58.16 ± 15.5	0.023*	0.51
State-Trait Anxiety Inventory (20–80)					
State anxiety	Before Training	35.13 ± 7.66	36.62 ± 3.13	0.764	0.07
	After Training	31.18 ± 5.84	36.93 ± 5.03	0.037*	0.65
Trait anxiety	Before Training	36.67 ± 3.91	34.37 ± 2.20	0.650	0.23
	After Training	31.98 ± 2.21	35.15 ± 3.71	0.024*	0.67

*p < 0.05; ^2^: Independent simple t test; *SD* Standart deviation; *OPG* Online Pilates group; *CG* Control group. *EPDS* Edinburgh Postnatal Depression Scale; *WDEQ-A* Wijma Delivery Expectancy/Experience Questionnaire A.

Significant values are in bold.

## Discussion

This study has shown that online Pilates training in pregnant women may reduce depression, anxiety, and fear of childbirth. Our study found that the depression levels of the participants in the Online Pilates group decreased, while the depression levels in the control group did not change. Although no study in the literature shows the effect of Pilates training on depression levels in pregnant women, studies show that regular exercise reduces depression levels in pregnant women^37,38^. In their meta-analysis study, Davenport et al.^39^ stated that exercise was at least as effective as psychological treatments in reducing prenatal depression. However, this effect did not continue in the postpartum period. They also emphasized that pregnant women should do at least 150 min of moderate-intensity exercise weekly for this effect to occur. Ji et al.^40^ pointed out the therapeutic effect of exercise in prenatal depression in their meta-analysis. They stated that yoga, gymnastics, aerobic exercise, and resistance exercises have effects on depression in pregnant women, but there is little literature on single exercise methods.

It is known from previous studies that regular exercise is effective in stress management, reduces depression, and improves personal self-esteem and body image^39,40^. Pilates training helped participants increase their self-confidence by increasing their awareness through principles such as concentration, breathing, and focus. Increased awareness plays a vital role in reducing the depressive moods of pregnant women. In addition, the fact that pregnant women communicated with other pregnant women through group exercise and thus did not feel lonely was effective in reducing depression levels. In addition, regular exercise may have made pregnant women feel more energetic, strong, and fit^41^ and may have reduced their depression. Davenport et al.^39^ stated that we arranged their exercises for at least two hours a week and at moderate intensity. As Ji et al.^40^ stated in their meta-analysis, we used Pilates exercises, which are aerobic, resistant exercise systems, and parameters such as flexibility and endurance, in our study. We think that Pilates has positive effects on depression, as it is a comprehensive exercise system that has all these characteristics and improves the body in every sense.

In our study, the anxiety levels of pregnant women decreased with Pilates training, but the anxiety level of the control group did not change. Studies have reported high anxiety levels during pregnancy, as was the case at the beginning of our study^30^. Aktan et al.^13^ stated that 8-week Pilates and birth training reduced the anxiety levels of pregnant women. In addition, other studies have shown that regular exercise positively affects stress management and anxiety^41,42^. The results of these studies on anxiety and exercise show similarities with our results. Reducing the level of anxiety will make a significant contribution to the pregnancy processes of pregnant women. The improvement in physical activity level, the physiological benefits of exercise, and its contribution to the mood of pregnant women may have effectively reduced anxiety. Pregnant women’s avoidance of both physical activity and social participation also causes a decrease in pregnant women’s communication with health professionals^9^. All of these may increase the anxiety levels of pregnant women. However, the fact that participants increase their social participation by exercising in small groups online and having the opportunity to consult a specialist about their problems may have reduced their anxiety levels.

While there was a decrease in the fear of birth of the participants in the Online Pilates group, there was no change in the control group. Sarpkaya Guder^43^, in her study with 108 pregnant, stated that Pilates and childbirth preparation training reduced the fear of childbirth. Pregnant Pilates training includes imagination, relaxation, and breathing techniques that can be done during childbirth. Considering the effects of Pilates training on the musculoskeletal system, all these techniques make the participants more comfortable and prepared for delivery. In addition, the diaphragm exercises used during Pilates can be used primarily in the pushing phase of labor, preparing pregnant women for birth^44^. It may improve the self-confidence of pregnant women, which is necessary for childbirth^14^. All these effects may have helped reduce the fear of childbirth in pregnant women.

Limited studies in the literature examine the effects of pilates exercises during pregnancy. The importance of online exercises is increasing, especially in recent years. Studies on this subject will guide the literature and clinicians. This is the most crucial strength of our study. Another strength is that the exercises are done weekly in small groups, with discipline, and accompanied by a physiotherapist. Our study has certain limitations. Limitations include the fact that pregnant women were not given birth education in addition to exercise, that the sample was not representative, and that pregnant women were not invited face-to-face for an interim check-up. There is a need for studies with larger samples comparing different methods.

## Conclusion

Online exercise methods can be used as reliable methods to improve the physical activity levels of pregnant women. This study shows the importance of online Pilates training for pregnant women to gain healthy habits and maintain social participation in all environments. Our study is a study that may guide the literature. Online Pilates training can reduce depression, anxiety, and fear of childbirth in pregnant women. Future studies need to examine the effects of online Pilates training in different groups. In addition, the effects of Online Pilates exercises with different types of online exercises or by adding birth education to the exercises need to be examined. The results of our study present online Pilates exercise training as a safe and applicable exercise method.

## Data Availability

The datasets generated during and/or analyzed during the current study are available from the corresponding author on reasonable request.

## References

[1] Davis EP, Narayan AJ (2020). Pregnancy as a period of risk, adaptation, and resilience for mothers and infants. Dev. Psychopathol..

[2] Tsakiridis I, Bakaloudi DR, Oikonomidou AC, Dagklis T, Chourdakis M (2020). Exercise during pregnancy: A comparative review of guidelines. J. Perinat Med..

[3] Dunkel Schetter C, Tanner L (2012). Anxiety, depression and stress in pregnancy: implications for mothers, children, research, and practice. Curr. Opin. Psychiatry..

[4] Johnson AR (2019). Fear of Childbirth among pregnant women availing antenatal services in a maternity hospital in rural Karnataka. Indian J. Psychol. Med..

[5] Alehagen S, Wijma B, Wijma K (2006). Fear of childbirth before, during, and after childbirth. Acta Obstet. Gynecol. Scand..

[6] Guney E, Unver H, Bal Z, Ucar T (2022). Psychosocial factors and health practices in pregnancy: A cross-sectional study. Int. J. Nurs. Pract..

[7] Melzer K (2010). Effects of recommended levels of physical activity on pregnancy outcomes. Am. J. Obstet. Gynecol..

[8] Evenson KR (2014). Guidelines for physical activity during pregnancy: Comparisons from around the world. Am. J. Lifestyle Med..

[9] Hillyard M, Sinclair M, Murphy M, Casson K, Mulligan C (2021). The impact of COVID-19 on the physical activity and sedentary behaviour levels of pregnant women with gestational diabetes. PLoS One.

[10] Bagherzadeh R (2021). Pregnancy; an opportunity to return to a healthy lifestyle: A qualitative study. BMC Pregn. Childbirth.

[11] Newton ER, May L (2017). Adaptation of maternal-fetal physiology to exercise in pregnancy: The basis of guidelines for physical activity in pregnancy. Clin. Med. Insights: Women’s Health.

[12] Birsner ML, Gyamfi-Bannerman C (2020). Physical activity and exercise during pregnancy and the postpartum period: ACOG committee opinion, Number 804. Obstet. Gynecol..

[13] Aktan B, Kayıkçıoğlu F, Akbayrak T (2021). The comparison of the effects of clinical Pilates exercises with and without childbirth training on pregnancy and birth results. Int. J. Clin. Pract..

[14] Ghandali NY, Iravani M, Habibi A, Cheraghian B (2021). The effectiveness of a Pilates exercise program during pregnancy on childbirth outcomes: A randomised controlled clinical trial. BMC Pregn. Childbirth..

[15] Janssen LE (2023). Stress-reducing interventions in pregnancy for the prevention of preterm birth: A systematic review and meta-analysis. J. Psychos. Obstet. Gynaecol..

[16] Mazzarino M, Kerr D, Morris ME (2018). Pilates program design and health benefits for pregnant women: A practitioners’ survey. J. Bodywork Mov. Ther..

[17] Perfeito RS, Allevato L, da Silva Silveira D (2019). Effects of the practice of Pilates in pregnancy: A literature review. Revista Saúde Física Mental.

[18] Doraiswamy S, Abraham A, Mamtani R, Cheema S (2020). Use of telehealth during the COVID-19 pandemic: Scoping review. J. Med. Internet Res..

[19] Bulguroglu HI, Bulguroglu M (2023). The effects of online pilates and face-to-face pilates in healthy individuals during the COVID-19 pandemic: A randomized controlled study. BMC Sports Sci. Med. Rehabil..

[20] Curtis RG, Ryan JC, Edney SM, Maher CA (2020). Can Instagram be used to deliver an evidence-based exercise program for young women? A process evaluation. BMC Public Health.

[21] Janjua S (2021). Digital interventions for the management of chronic obstructive pulmonary disease. Cochrane Database Syst. Rev..

[22] Gungor F, Tarakci E, Ozdemir-Acar Z, Soysal A (2022). The effects of supervised versus home Pilates-based core stability training on lower extremity muscle strength and postural sway in people with multiple sclerosis. Mult. Scler. J..

[23] da Ortega-Pérez Villar L (2020). Comparison of intradialytic versus home-based exercise programs on physical functioning, physical activity level, adherence, and health-related quality of life: Pilot study. Sci. Rep..

[24] Suner-Keklik S, Numanoglu-Akbas A, Cobanoglu G, Kafa N, Guzel NA (2022). An online pilates exercise program is effective on proprioception and core muscle endurance in a randomized controlled trial. Ir. J. Med. Sci..

[25] Hyun AH, Cho JY, Koo JH (2022). Effect of home-based tele-pilates intervention on pregnant women: A pilot study. Healthcare.

[26] Kim HB, Hyun AH (2022). Psychological and biochemical effects of an online pilates intervention in pregnant women during COVID-19: A randomized pilot study. Int. J. Environ. Res. Public Health..

[27] Cox JL, Holden JM, Sagovsky R (1987). Detection of postnatal depression. Development of the 10-item Edinburgh postnatal depression scale. Br. J. Psychiatry..

[28] Bunevicius A, Kusminskas L, Pop VJ, Pedersen CA, Bunevicius R (2009). Screening for antenatal depression with the Edinburgh depression scale. J. Psychosom. Obstet. Gynaecol..

[29] Muskens L (2022). The association of unplanned pregnancy with perinatal depression: A longitudinal cohort study. Arch Womens Ment Health..

[30] Lebel C, MacKinnon A, Bagshawe M, Tomfohr-Madsen L, Giesbrecht G (2020). Elevated depression and anxiety symptoms among pregnant individuals during the COVID-19 pandemic. J. Affect. Disord..

[31] Engindeniz, A.N., Kuey, L., & Kultur, S. *Turkish version of the Edinburgh Postpartum Depression Scale. Reliability and validity study.* Spring Symposiums I book. Psychiatric Organization of Turkey, Ankara. (1996).

[32] Spielberger, C.D., Gorsuch, R.L., Lushene, R., Vagg, P.R. & Jacobs, G.A. *Manual for the State-Trait Anxiety Inventory STAI (Form Y).* (Palo Alto, 1983)

[33] Wijma K, Wijma B, Zar M (1998). Psychometric aspects of the W-DEQ; a new questionnaire for the measurement of fear of childbirth. J Psychosom. Obstet. Gynaecol..

[34] Oner N, Le Compte A (1982). Handbook of the State-Trait Anxiety Inventory.

[35] Korukcu O, Kukulu K, Firat MZ (2012). The reliability and validity of the Turkish version of the Wijma delivery expectancy/experience questionnaire (W-DEQ) with pregnant women. J. Psychiatr. Ment. Health Nurs..

[36] Cobanoglu G (2019). The relationship between scapular and core muscle endurance in Professional athletes. Ann. Med. Res..

[37] Morres ID (2022). Exercise for perinatal depressive symptoms: A systematic review and meta-analysis of randomized controlled trials in perinatal health services. J. Affect Disord..

[38] Zhu Y (2021). The effect of music, massage, yoga and exercise on antenatal depression: A meta-analysis. J. Affect Disord..

[39] Davenport MH (2018). Impact of prenatal exercise on both prenatal and postnatal anxiety and depressive symptoms: A systematic review and meta-analysis. Br. J. Sports Med..

[40] Ji M, Li R, Xu Y (2024). Meta-analysis of the effect of different exercise modalities in the prevention and treatment of perinatal depression. J. Affect. Disord..

[41] Roh SY (2016). Effect of a 16-week Pilates exercise program on the ego resiliency and depression in elderly women. J. Exerc. Rehabil..

[42] Kołomańska-Bogucka D, Micek A, Mazur-Bialy AI (2022). The COVID-19 pandemic and levels of physical activity in the last trimester, life satisfaction and perceived stress in late pregnancy and in the early puerperium. Int. J. Environ. Res. Public Health..

[43] Guder DS, Yalvac M, Vural G (2018). The effect of pregnancy Pilates-assisted childbirth preparation training on childbirth fear and neonatal outcomes: A quasi-experimental/quantitative research. Quality Quantity..

[44] Sarpkaya-Guder D (2018). Pregnancy Pilates and benefts of pregnancy pilates during childbirth. Yoga Phys. Ther. Rehabil..

